# Multimodal radiomics fusion for predicting postoperative recurrence in NSCLC patients

**DOI:** 10.1007/s00432-025-06311-w

**Published:** 2025-09-18

**Authors:** Ghazal Mehri-kakavand, Sibusiso Mdletshe, Mehdi Amini, Alan Wang

**Affiliations:** 1 Department of Anatomy and Medical Imaging, Faculty of Medical and Health Sciences, University of Auckland, Auckland, New Zealand; 2 Division of Nuclear Medicine and Molecular Imaging, Geneva University Hospital, Geneva, Switzerland; 3 Auckland Bioengineering Institute, The University of Auckland, Auckland, New Zealand; 4 Centre for Brain Research, The University of Auckland, Auckland, New Zealand; 5 Matai Medical Research Institute, Gisborne, , New Zealand; 6 Medical Imaging Research Centre, The University of Auckland, Auckland, New Zealand; 7 Centre for Co-Created Ageing Research, The University of Auckland, Auckland, New Zealand

**Keywords:** Non-small cell lung cancer, Postoperative recurrence, Radiomics, Multimodal fusion, PET/CT integration, Machine learning

## Abstract

**Purpose:**

Postoperative recurrence in non-small cell lung cancer (NSCLC) affects up to 55% of patients, underscoring limits of TNM staging. We assessed multimodal radiomics—positron emission tomography (PET), computed tomography (CT), and clinicopathological (CP) data—for personalized recurrence prediction.

**Methods:**

Data from 131 NSCLC patients with PET/CT imaging and CP variables were analysed. Radiomics features were extracted using PyRadiomics (1,316 PET and 1,409 CT features per tumor), with robustness testing and selection yielding 20 CT, 20 PET, and 23 CP variables. Prediction models were trained using Logistic Regression (L1, L2, Elastic Net), Random Forest, Gradient Boosting, XGBoost, and CatBoost. Nested cross-validation with SMOTE addressed class imbalance. Fusion strategies included early (feature concatenation), intermediate (stacked ensembles), and late (weighted averaging) fusion.

**Results:**

Among single modalities, CT with Elastic Net achieved the highest cross-validated AUC (0.679, 95% CI: 0.57–0.79). Fusion improved performance: PET + CT + Clinical late fusion with Elastic Net achieved the best cross-validated AUC (0.811, 95% CI: 0.69–0.91). Out-of-fold ROC curves confirmed stronger discrimination for the fusion model (AUC = 0.836 vs. 0.741 for CT). Fusion also showed better calibration, higher net clinical benefit (decision-curve analysis), and clearer survival stratification (Kaplan–Meier).

**Conclusion:**

Integrating PET, CT, and CP data—particularly via late fusion with Elastic Net—enhances discrimination beyond single-modality models and supports more consistent risk stratification. These findings suggest practical potential for informing postoperative surveillance and adjuvant therapy decisions, encouraging a shift beyond TNM alone toward interpretable multimodal frameworks. External validation in larger, multicenter cohorts is warranted.

**Supplementary Information:**

The online version contains supplementary material available at 10.1007/s00432-025-06311-w.

## Introduction

Non-small cell lung cancer (NSCLC) accounts for over 85% of lung cancer cases and remains the leading cause of cancer-related mortality worldwide (Sasaki et al. 2022). Despite advances in diagnosis and treatment, postoperative recurrence rates remain high, with 30–55% of patients experiencing relapse within five years (Uramoto and Tanaka 2014; D’Antonoli et al. 2020). Recurrence leads to increased treatment costs, additional interventions, diminished quality of life, and reduced survival, imposing substantial burdens on patients and healthcare systems (Uramoto and Tanaka 2014; Vachon et al. 2021). Early and accurate prediction of recurrence is essential to improve patient outcomes by tailoring personalized therapies (Taiichi Wakiya et al. 2022), timely intervention in high-risk patients and avoiding overtreatment in low-risk cases (Johnson et al. 2022). Traditional methods, such as TNM staging, focus solely on the anatomical characteristics of the tumor, neglecting the complex biological and molecular heterogeneity of cancer (Lococo et al. 2024 ). This limitation results in patients with similar stages experiencing vastly different outcomes (Song et al. 2020). These challenges underscore the need for advanced approaches that integrate biological and clinical data to enhance the precision of recurrence risk assessments and tumor behavior predictions (Sanvito et al. 2021).

Radiomics enables the extraction of complex quantitative features from imaging modalities such as computed tomography (CT) and positron emission tomography (PET) (Guiot et al. 2022). These features reflect histological traits, tumor shape, intensity, and heterogeneity, revealing subtle details beyond human perception (Song et al. 2023). For instance, radiomics can detect variations in tissue density or tumor heterogeneity missed by conventional imaging (Antunes et al. 2022). However, comprehensive tumor characterization requires integration of radiomics with biological and clinical data (Grossmann et al. 2017). Artificial intelligence (AI), particularly machine learning, facilitates analysis of complex, non-linear radiomic data to build advanced predictive models. These methods uncover patterns linked to clinical outcomes and aid discovery of novel biomarkers for cancer progression and recurrence (Parmar et al. 2015; Ahn et al. 2019; Christie et al. 2021; Kim et al. 2022; Tabassum et al. 2023).

Integrating multimodal data—combining PET and CT imaging features with clinicopathological information—has improved predictive accuracy for NSCLC recurrence (Ahn et al. 2019; Lv et al. 2019; Christie et al. 2021; Kim et al. 2022). Multiple studies demonstrate that multimodality approaches outperform single-modality models (D’Antonoli et al. 2020; Moon et al. 2020; Bove et al. 2023). For example, Christie et al. combined tumor and peritumoral radiomic features from CT with distant bone marrow metastasis features from FDG PET, along with clinical data, to stratify patients into recurrence risk groups. Their model outperformed staging alone (Christie et al. 2022). Wang et al. showed that CT radiomics integrated with clinical data enhances recurrence risk prediction beyond either model alone (Wang et al. 2024). Similarly, Akinci D'Antonoli et al. found that adding radiomics signatures from tumoral and peritumoral regions to pTNM staging improved prediction accuracy over pTNM alone (D’Antonoli et al. 2020). Bove et al. employed transfer learning with CNNs to fuse CT images and clinical data from tumor and peritumoral regions, achieving notable gains in recurrence prediction accuracy (Bove et al. 2023). Together, these findings underscore the value of multimodal data integration for robust prediction.

Most NSCLC recurrence prediction studies have focused on CT alone or combined with clinical data, with few incorporating PET, CT, and clinicopathological information together. Moreover, limited research has explored different fusion techniques or compared diverse machine learning classifiers to determine the optimal approach. This study fills these gaps by assessing multiple fusion strategies—feature- and decision-level fusion—and systematically comparing various classifiers across single and combined modalities, providing comprehensive insights to improve predictive accuracy and advance precision oncology.

## Material and methods

### Data collection

This study analysed preoperative PET and CT scans along with clinicopathological data from 211 NSCLC patients in The Cancer Imaging Archive (TCIA) NSCLC-RADIOGENOMICS—The Cancer Imaging Archive (TCIA) (Bakr et al. 2018). All patients underwent tumor resection, with recurrence monitored over eight years. The dataset consists of two cohorts: R01 (162 patients, mean age 68.6) from Stanford University and Palo Alto VA (2008–2012), and AMC (49 patients, mean age 65.8) from Stanford University. This study focused on the R01 cohort, which includes tumor segmentation masks, CT and PET images, and clinical data. After excluding 31 patients for missing masks or poor image quality, 131 patients remained, of whom 38 (29%) experienced recurrence. Imaging parameters are detailed in the Bakr et al. study (Bakr et al. 2018).

### Data preparation

#### Clinicopathological data

Clinicopathological variables included age, weight, gender, tumor histology, pathological TNM stage, histological grade, lymphovascular and pleural invasion, ground-glass opacity percentage, ethnicity, smoking status, and pack-years. Categorical variables were one-hot encoded, resulting in 27 features. The primary outcome was the binary endpoint High_Risk_2yr (1 = recurrence within 2 years, 0 = no recurrence or censored). In addition, traditional survival variables (Event, defined as recurrence during the ~ 8-year follow-up, and Survival_Time in days) were retained solely for Kaplan–Meier survival analysis and log-rank testing.

#### Image preprocessing

**Fig. 1:**
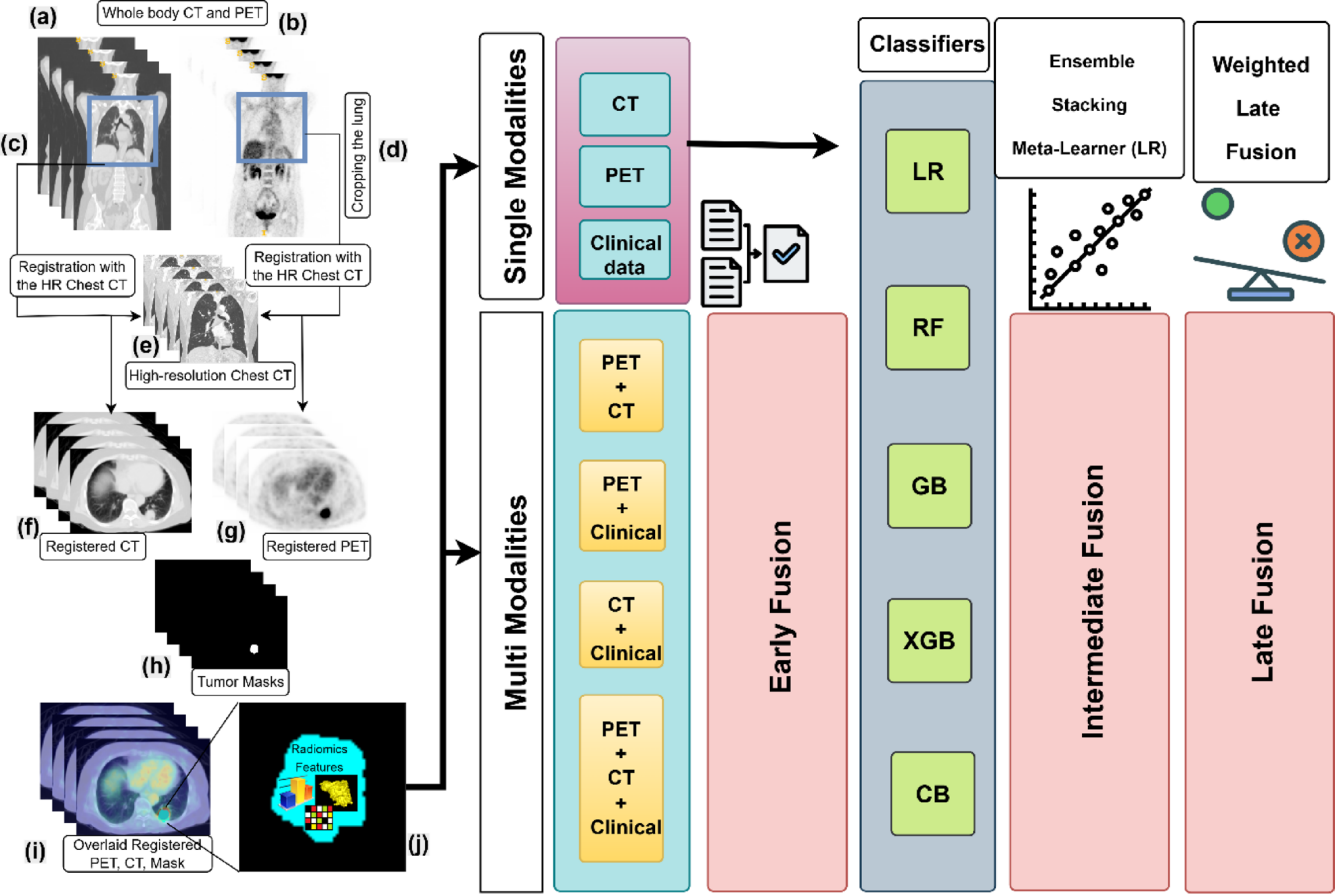
Study pipeline. **a** Whole body CT, **b** whole body PET, **c** cropped CT, **d** cropped PET, **e** chest CT HR, **f** registered CT, **g** registered PET, **h** mask, **i** overlaid registered PET_CT_Mask, **j** radiomic features. NSCLC_Radiogenimics-R01 dataset, Case_ID:55, Slice NO: 241. Extracted features from CT, PET, and clinicopathological data were used individually or in fusion (early, intermediate, late) with multiple classifiers (LR: logistic regression, RF: random forest, GB: gradient boosting, XGB: XGBoost, CB: CatBoost). Model evaluation included ROC-AUC, calibration, decision-curve analysis, and survival stratification

Images were converted from DICOM to NIfTI format using MRIcroGL (v1.2.20220720) and 3D Slicer (v5.6.2). Lung region cropping, co-registration, and resampling to isotropic 1 mm^3^ voxels were performed with 3D Slicer (v5.6.2) and ITK-SNAP (v2.4.0) to ensure spatial consistency for feature extraction. PET intensities were standardized to Standardized Uptake Values (SUV) using DICOM metadata, while CT values—already in Hounsfield units—required no further standardization. Binary segmentation masks were resampled to align with registered images using nearest-neighbor interpolation in ITK-SNAP (v2.4.0) (Fig. 1).

#### Feature extraction and selection

Radiomics features were extracted from both PET and CT images using the open-source PyRadiomics package (version 3.1.0) in Python (version 3.8.19). The source code for this library is available on GitHub—AIM-Harvard/pyradiomics. This library yielded 1,316 PET and 1,409 CT features per tumor region. Redundant features were removed using Pearson correlation (threshold > 0.95), as supported by previous radiomics research (Wang et al. 2019; Yang et al. 2020). Feature selection was performed using the ANOVA F-test, as it is commonly used in radiomics studies (Ober Van Gómez et al. 2022; Sevinj Yolchuyeva et al. 2023 ; Wenchao Zhang et al. 2023). Recursive feature elimination (RFE) with Logistic Regression (max iterations = 1000) ranked features by iteratively removing the least predictive to improve model interpretability and performance (Perniciano et al. 2024). A z-score transformation was then applied to CT and PET features to ensure standardization.

### Model development

We implemented a comprehensive machine-learning framework to evaluate multiple classifiers and modality combinations for postoperative recurrence prediction. A total of five classifiers were examined: (i) logistic regression with both L2 and ElasticNet regularization variants (the latter using the *saga* solver with a maximum of 2000 iterations and class balancing), (ii) random forest, (iii) gradient boosting (scikit-learn implementation), (iv) XGBoost with a logistic objective and log-loss evaluation metric, (v) CatBoost with an AUC-based objective (when available).

All classifiers were trained using a nested stratified cross-validation (CV) design to ensure unbiased performance estimates. The outer loop employed a three-fold stratified CV, which provided out-of-fold (OOF) predictions for the entire cohort and served as the basis for unbiased evaluation. Within each outer training fold, an inner loop consisting of two-fold stratified CV was used to perform randomized hyperparameter search (five iterations). The best configuration identified in the inner loop was re-trained on the full outer training set, after which probabilistic outputs were calibrated using isotonic regression through a prefitted CalibratedClassifierCV. This procedure ensured that model predictions were both optimized and well-calibrated before evaluation on the corresponding outer test set.

All classifiers used a single imbalanced-learn Pipeline to standardize preprocessing and prevent leakage: (1) drop features with > 50% missingness, near-zero variance (< 1 × 10⁻⁸), or non-finite values; (2) impute remaining missing values via k-NN (k = 5) or median (fast runs); (3) remove highly correlated clinical variables (|r|> 0.95); (4) apply robust scaling to continuous clinical covariates (Age, Weight, Pack-years); and (5) apply SMOTE (k = 3) within training folds to address class imbalance. The strategy was run across seven modality configurations: CT, PET, Clinical; PET + CT, PET + Clinical, CT + Clinical; and PET + CT + Clinical. Primary evaluation used ROC AUC with 95% CIs estimated by DeLong. Secondary metrics (accuracy, balanced accuracy, precision, recall, F1) were computed from out-of-fold predictions. Confusion matrices at clinically chosen thresholds characterized sensitivity–specificity trade-offs. Model calibration was evaluated using reliability curves, the Brier score, and expected calibration error (ECE). Between-model differences in ROC AUC were tested with pairwise DeLong comparisons across classifiers and modality combinations.

#### Fusion strategy development

Beyond single-modality models, we evaluated three fusion paradigms—feature-level (early), intermediate (stacked/meta-learning), and decision-level (late)—to integrate complementary CT, PET, and clinical information. Early fusion concatenated modality-specific feature sets into a single design matrix with modality prefixes (e.g., PET:, CT:, Clinical:), preserving provenance and enabling joint optimization within one pipeline. Class imbalance was addressed within cross-validation using SMOTE.

Intermediate fusion used stacked generalization: base models produced OOF probabilities per modality; meta-features (mean, max, min, SD, Shannon entropy) were concatenated and fed to a class-weighted logistic-regression meta-learner with isotonic calibration. Late fusion aggregated probabilities from the best single-modality models via optimized modality weights (chosen to maximize cross-validated ROC AUC), rather than simple averaging. Fused outputs were assessed within the same nested-CV framework using ROC AUC/PR AUC and threshold-based metrics.

### Statistical significance testing

We used DeLong’s test for correlated ROC curves (α = 0.05) on out-of-fold predictions from nested CV. Two comparison levels were performed: (1) cross-combination (best late-fusion vs best single-modality) and (2) within-combination (pairwise classifiers trained on the same modality/fusion set). Significance was set at *p* < 0.05. Holm adjustment was optionally applied within each comparison family; primary results report unadjusted *p*-values.

### Decision curve analysis

Decision curve analysis (DCA) was performed to evaluate the clinical utility of the predictive models. Specifically, DCA was applied to the best-performing single-modality model (CT/ElasticNet) and the best-performing fusion model (PET + CT + Clinical/ElasticNet). Out-of-fold predicted probabilities from nested cross-validation were used to compute net benefit across a clinically relevant range of threshold probabilities (0.05–0.30). For each threshold, the net benefit of the model was compared against default strategies of treating all or treating none. The resulting curves were plotted with a shaded region highlighting the clinically plausible range (0.10–0.30).

### Survival analysis

We assessed prognostic utility by linking 2-year recurrence predictions to long-term outcomes. Models trained for the binary endpoint (recurrence ≤ 730 days) were evaluated over ~ 8 years by stratifying patients into high-/low-risk groups based on predicted probabilities. Candidate cut-offs (0.10, 0.15, 0.20, 0.30) were screened; the cut-off with the smallest log-rank *p*-value defined the strata. Kaplan–Meier curves with 95% CIs and two-sided log-rank tests quantified differences in recurrence-free survival. Final plots report group sizes, numbers at risk, threshold, and *p*-value.

### Implementation

Implemented end-to-end in Python (scikit-learn, XGBoost, CatBoost) with parallel processing. Analyses produced standardized artifacts—ROC curves, confusion matrices, calibration and survival plots—supporting reproducibility and clinical interpretability.

## Results

### Patient cohort characteristics

Among 131 patients, 29.0% (38/131) experienced recurrence. Mean age was 69.1 ± 8.6 years (47.3% aged 60–70); most were male (76.3%) and Caucasian (74.8%). Smoking exposure was high (35.2 ± 29.8 pack-years; 38.2% > 40); 64.9% were former, 20.6% current, and 14.5% never smokers. Adenocarcinoma predominated (78.6%; squamous 19.1%). Early stage was common (N0 79.4%; M0 96.9%); grade was most often moderate (47.3%). Adverse pathology was infrequent (lymphovascular invasion 13.7%; pleural invasion 28.2%). Survival: mean 1092.1 ± 781.9 days; median 1062.0 (IQR 300.0–1826.5); range 6–3074. Comprehensive characteristics are summarized in supplementary table (Table S1).

### Feature extraction and selection

Following preprocessing and filtering, 20 radiomics features from CT, 20 from PET, and 23 clinical variables were retained for analysis. These features were used as inputs to subsequent modeling and fusion strategies.

### Model development results

#### Early fusion (feature-level fusion)

In early fusion, ElasticNet performed best. CT + Clinical achieved the highest AUC at 0.725 (95% CI 0.609–0.841), followed by PET + CT + Clinical at 0.714 (0.593–0.835). CT alone with ElasticNet reached 0.679, whereas PET-only was weaker (0.576). Pairwise DeLong tests showed ElasticNet outperforming RandomForest (CT, Clinical, PET + CT + Clinical) and exceeding XGBoost (CT) and GradientBoosting (Clinical) (all *p* < 0.05).

#### Intermediate (stacking) and late (score-level) fusion

We evaluated score- and meta-level fusion across all paired and tri-modal sets. For late fusion, we combined calibrated modality probabilities via simple averaging, hard voting, and optimized weighted averaging (tuned for ROC AUC or PR AUC). AUC-optimized weighting performed best: in PET + CT + Clinical with ElasticNet bases it achieved AUC = 0.811 (95% CI 0.742–0.879) and PR-AUC = 0.581, outperforming single-modality and pairwise fusions. For stacking (intermediate fusion), meta-features from calibrated probabilities (mean, max, min, SD, entropy) trained a logistic-regression meta-learner with isotonic calibration. Although stacking improved stability for some bases, it was consistently inferior to weighted late fusion, especially in the tri-modal setting.

#### Decision curve analysis (DCA) for single and multimodal models

**Fig. 2:**
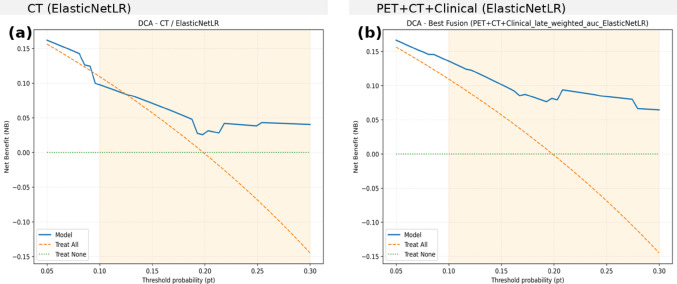
Decision curve analysis (DCA) for recurrence prediction: **a** CT/ElasticNetLR and **b** PET + CT + Clinical/ElasticNetLR. Both models provided positive net benefit across clinically relevant thresholds (0.05–0.30), with the fusion model showing consistently higher net benefit than CT alone

Decision curve analysis (DCA) was performed to assess the clinical utility of the best single-modality model (CT/ElasticNetLR) and the best fusion model (PET + CT + Clinical/ElasticNetLR). Both models demonstrated positive net benefit across a clinically relevant threshold range (0.05–0.30), compared with default strategies of “treat all” or “treat none.” For the CT-only model, net benefit was modest and declined steadily with increasing threshold probability, approaching the “treat all” strategy near 0.20. In contrast, the PET + CT + Clinical fusion model provided consistently higher net benefit across the same range, maintaining a clear advantage over both default strategies and the CT-only model. The gain was most pronounced between thresholds of 0.10 and 0.20, corresponding to plausible clinical decision points. Figure 2 presents the results of the decision curve analyses (DCAs).

### Survival analysis

**Fig. 3:**
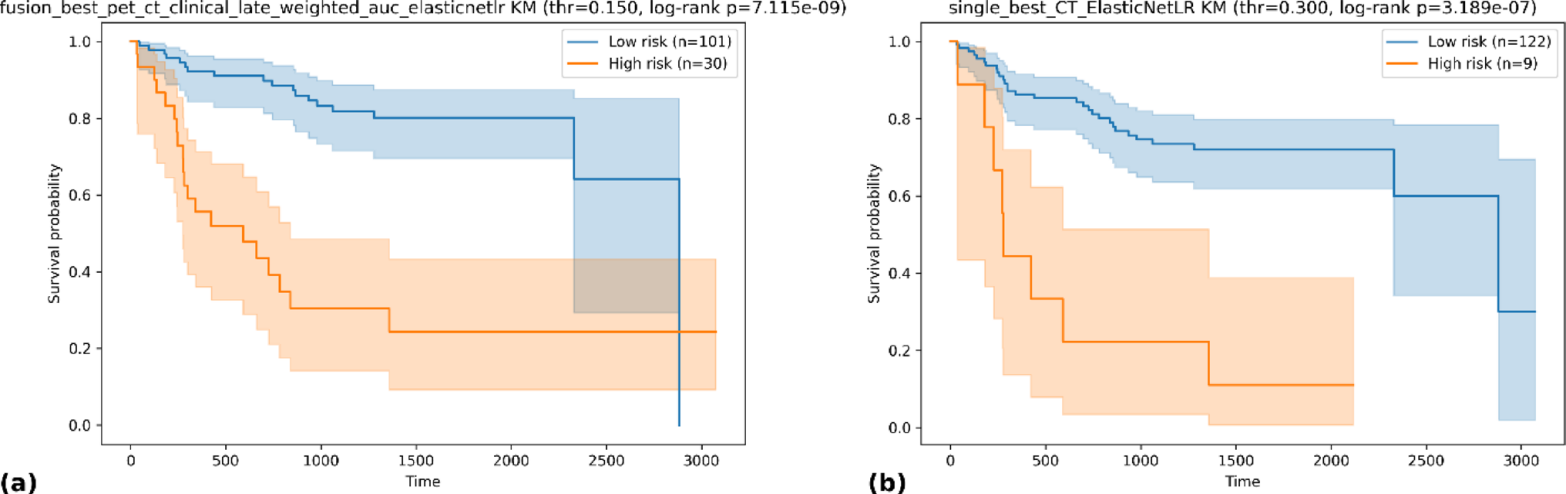
Kaplan–Meier recurrence-free survival curves stratified by predicted 2-year recurrence risk: **a** PET + CT + Clinical/ElasticNetLR and **b** CT/ElasticNetLR. Both models significantly separated high- and low-risk groups, with the fusion model achieving clearer stratification (log-rank *p* = 7.1 × 10^−9^ vs. 3.2 × 10^−7^)

Kaplan–Meier analysis evaluated whether 2-year recurrence probabilities yielded long-term separation. Patients were dichotomized at model-specific cutoffs (PET + CT + Clinical/ElasticNet 0.15; CT/ElasticNet 0.30). Fusion classified 30 high-risk vs 101 low-risk, with early and sustained separation over ~ 8 years (log-rank p = 7.1 × 10⁻⁹). CT-only classified 9 vs 122 with significant separation (p = 3.2 × 10⁻⁷). Both models stratified risk, but fusion produced a larger, more balanced high-risk group and more stable estimates (Fig. 3).

### Calibration analysis

Probability calibration was evaluated using the Brier score and expected calibration error (ECE). Both models showed close agreement between predicted and observed recurrence risk. The fusion model showed slightly better calibration, reflected by a lower Brier score (0.158 vs. 0.173) while ECE was identical for both (0.084).

### Interpretability and feature importance

To better understand model decision-making, we assessed feature importance using coefficients, permutation analysis, and SHAP values for the best-performing models in each modality and for the multimodal fusion model. For the CT-only model (ElasticNetLR), recurrence predictions were primarily driven by first-order intensity features, with skewness emerging as the most influential variable across coefficients, permutation analysis, and SHAP values. Mean intensity and maximum intensity also contributed, alongside entropy-based descriptors.

**Fig. 4:**
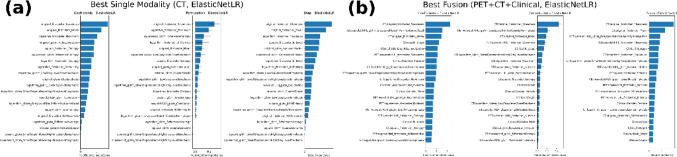
Feature importance for recurrence prediction using ElasticNetLR: **a** CT-only model, where first-order intensity features (skewness, mean, maximum) dominated; **b** PET + CT + Clinical fusion model, where CT skewness remained most influential, complemented by PET texture and CT mean intensity

In the PET + CT + Clinical fusion model (ElasticNetLR), CT skewness remained the dominant predictor, complemented by CT mean intensity and a PET wavelet-transformed texture feature (GLRLM GrayLevelNonUniformityNormalized), indicating that both intensity-based and metabolic heterogeneity markers shaped recurrence risk estimation (Fig. 4).

### Discrimination performance (ROC–AUC and confusion matrices)

**Fig. 5:**
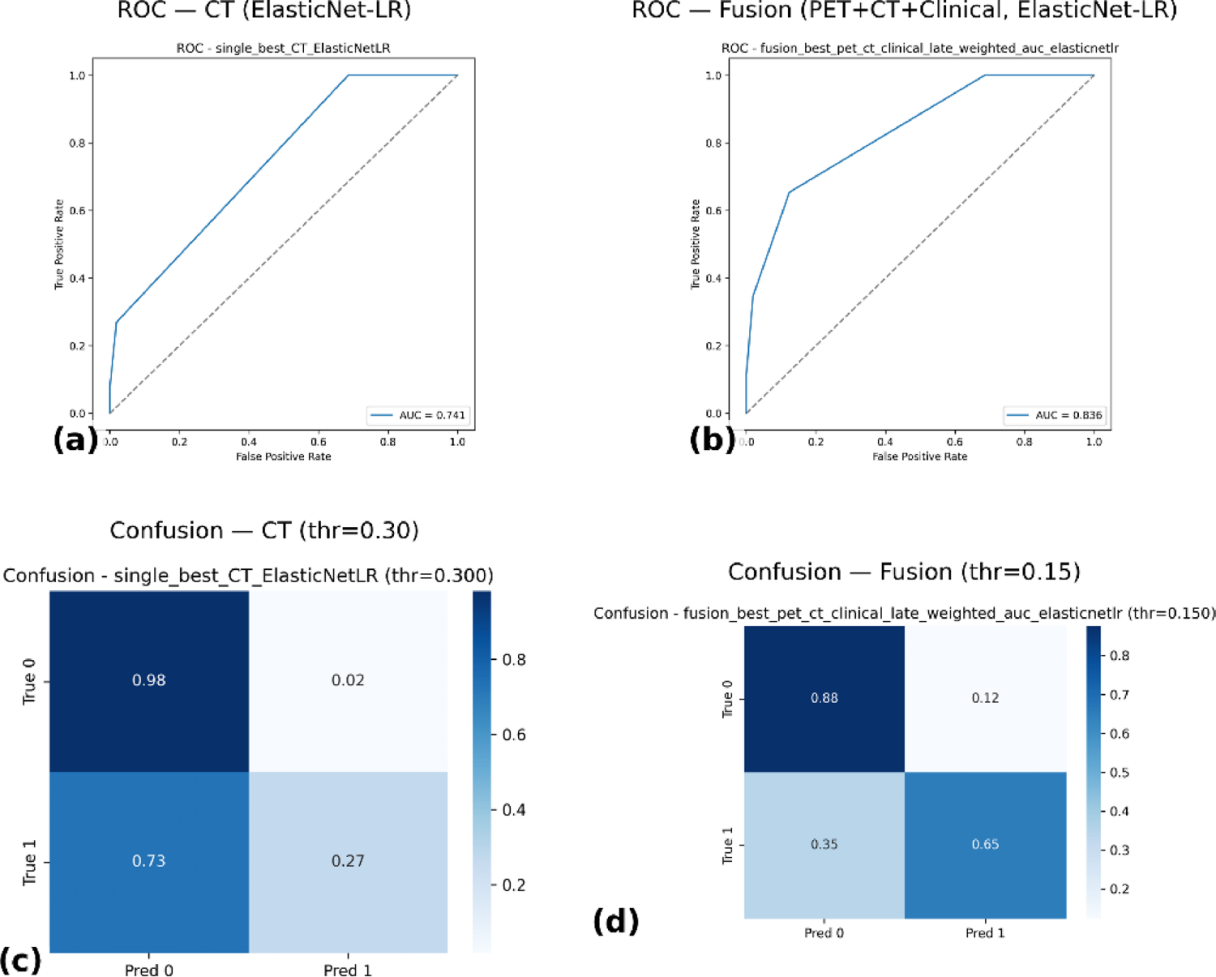
Comparative discrimination and operating performance of the best single-modality and fusion models

Nested CV showed CT/ElasticNetLR AUC 0.679 (95% CI 0.57–0.79) versus PET + CT + Clinical/ElasticNetLR 0.714 (0.59–0.84). OOF ROC–AUC rose from 0.741 (CT) to 0.836 (fusion). At operating thresholds, CT at 0.30 had high specificity (0.98) but low sensitivity (0.27; F1 = 0.40), whereas fusion at 0.15 balanced specificity 0.88 and sensitivity 0.65 (F1 = 0.61). Overall, late fusion improved discrimination and delivered a more clinically useful sensitivity–specificity trade-off (Table 1, Fig. 5).

**Table 1:** Performance of the best single-modality (CT/ElasticNetLR) and fusion model (PET + CT + Clinical/ElasticNetLR) under NCV and OOF evaluation, showing superior discrimination and balance for fusion

Model	Source	Threshold	ROC–AUC (95% CI)	PR–AUC	Accuracy	Balanced Acc	Precision	Recall (Sensitivity)	Specificity	F1
CT/ElasticNetLR	NCV*	0.5 (default)	0.679 (0.57–0.79)	–	0.840	0.625	0.778	0.269	–	0.400
PET + CT + Clinical (late, ElasticNetLR)	NCV	0.5 (default)	0.714 (0.59–0.84)	–	0.817	0.625	0.571	0.308	–	0.400
CT/ElasticNetLR	OOF*	0.30	0.741	0.42	0.840	0.625	0.778	0.269	0.981	0.400
PET + CT + Clinical (late, ElasticNetLR)	OOF	0.15	0.836	0.57	0.832	0.765	0.567	0.654	0.876	0.607

*NCV = Nested Cross-Validation, reporting mean performance across inner/outer folds with default threshold (0.5)

*OOF = Out-of-Fold evaluation, reporting performance on held-out data across folds at the operating thresholds chosen for optimal balance of sensitivity and specificity

### DeLong significance testing

Late-fusion AUCs were numerically higher than single-modality results, but cross-combination DeLong tests showed no significant advantage for PET + CT + Clinical over the best single baseline (α = 0.05). Within-combination pairwise tests yielded significant differences in CT (n = 6), CT + Clinical (n = 2), Clinical (n = 4), PET (n = 1), and PET + CT + Clinical (n = 6). Across these, ElasticNetLR consistently outperformed weaker classifiers (e.g., RandomForest, stacking), with additional wins over GradientBoosting, XGBoost, and CatBoost (all p < 0.05).

## Discussion

### Principal findings

This study demonstrates that integrating CT, PET, and clinicopathologic variables via ElasticNet-based late fusion provided the strongest overall performance for predicting postoperative recurrence in early-stage NSCLC. CT radiomics was the best single modality, consistent with the prognostic value of CT-derived heterogeneity. Late fusion achieved the highest discrimination (AUC = 0.811), improved calibration, and superior clinical utility on decision-curve analysis. Kaplan–Meier analyses showed clearer, more durable separation of fusion-defined risk groups than CT alone, highlighting complementary contributions from imaging and clinical data.

### Comparative performance of early, intermediate, and late fusion approaches

Multimodal integration improved recurrence prediction in NSCLC. Early fusion showed clear gains from combining CT radiomics with clinical variables, while PET contributed only modest incremental signal in this cohort. ElasticNet consistently outperformed tree-based learners, reflecting its stability and capacity for regularization rather than reliance on model complexity. CT-alone models achieved reasonable discrimination but favoured specificity at the expense of sensitivity, limiting clinical utility in contexts where missing high-risk patients has significant consequences. By contrast, PET + CT + Clinical late fusion delivered a more balanced sensitivity–specificity profile and a higher F1 score, better aligning with clinical priorities such as identifying candidates for adjuvant therapy.

Stacking ensemble (intermediate fusion) provided more stable performance across folds but was less accurate than weighted late fusion. This underperformance may reflect several factors: limited diversity among base learners, overfitting of the logistic regression meta-learner given the modest cohort size, and the relatively weak contribution of certain base classifiers. Together, these issues likely reduced the meta-learner’s ability to extract complementary signal. In larger, more heterogeneous datasets, stacking may regain its advantage, but within our cohort, optimized weighted late fusion proved more effective. Weighted late fusion achieved the best overall performance, yielding the highest AUC and PR-AUC in the tri-modal PET + CT + Clinical setting. These findings underscore the complementary value of integrating metabolic, morphological, and clinical features, and support calibrated late fusion as the most reliable and clinically relevant approach.

Our findings align with prior studies that showed incremental value of combining radiomics with clinical staging. Amini et al. (2022), Lian et al. (2022), and Wang et al. (2024) showed that integrating clinical and radiomics data can enhance model performance. Moon et al. (2020) reported that integrating CT radiomics with TN stage improved recurrence prediction compared with TN staging alone, though the gain was modest and statistical significance was limited. Similarly, D’Antonoli et al. (2020) demonstrated that tumoral plus peritumoral CT radiomics signatures enhanced risk stratification beyond TNM stage. In contrast, the study conducted by Kirienko et al. (2018) found that adding clinical data did not lead to a significant improvement in predictive performance. More recent deep-learning approaches by Shimada et al. (2022) and Sasaki et al. (2022) also highlighted the prognostic potential of CT radiomics and AI-extracted features in early-stage disease. Yet, most of these studies excluded PET data despite its established biological relevance. Christie et al. (2022) and Kim et al. (2022) are notable exceptions, incorporating PET-CT and multimodal features to achieve higher performance in prediction of NSCLC recurrence.In another study of head and neck cancer, Salmanpour et al. (2021) highlighted the efficacy of hybrid machine learning systems incorporating PET–CT fusion strategies, demonstrating significant improvements in predicting overall survival and distant metastasis.Our study builds on this trajectory by explicitly comparing unimodal and multimodal strategies within a nested cross-validation framework and by quantifying net clinical benefit with decision-curve analysis, which was absent in most prior work.

### Clinical implications

#### Decision-curve analysis

DCA indicates greater clinical utility for multimodal fusion than CT radiomics alone. CT offered modest benefit at low thresholds but waned with increasing thresholds, whereas PET + CT + Clinical maintained consistently higher net benefit—reducing unnecessary interventions in low-risk patients while identifying more high-risk cases. The advantage was most evident at 0.10–0.20, a plausible range for adjuvant therapy or intensified surveillance. Both models outperformed “treat none,” but only fusion consistently exceeded “treat all,” supporting its use for risk-adapted management in NSCLC.

#### Kaplan–Meier stratification

Models trained for 2-year recurrence yielded durable prognostic separation over ~ 8 years: patients flagged “high risk” showed persistently worse recurrence-free survival. Modality choice affected strata: CT/ElasticNet identified a smaller high-risk group with sharper separation but wider CIs, whereas PET + CT + Clinical classified a larger high-risk subset, producing clearer, more stable long-term stratification. These patterns suggest multimodal fusion better captures underlying disease biology and generalizes more robustly. Overall, model-derived probabilities have prognostic value and PET/CT + clinical fusion supports individualized postoperative risk stratification.

#### Interpretability and feature importance

These results highlight the complementary prognostic value of imaging-derived and clinical predictors. In the CT-only setting, skewness and related intensity feature captured tumor asymmetry and irregularity, supporting their role as robust radiomic biomarkers of recurrence.This aligns with prior evidence suggesting that intensity-based (first-order) features, which characterize the distribution of voxel intensities within the tumor area and reflect grayscale pixel values, play a crucial role in CT imaging (Florez et al., 2018). In line with our findings, Zaman et al. (2024) further demonstrated that first-order features extracted from CT images, were highly predictive of clinical outcomes, emphasizing the potential of intensity-based metrics in survival prediction models. In the fusion model, CT features continued to dominate, while PET texture descriptors introduced metabolic heterogeneity, broadening the biological scope of the predictions.Consistent with this, Pellegrino et al. (2024) demonstrated that PET-derived radiomics features place a stronger emphasis on texture characteristics. Similarly, studies by Piñeiro-Fiel et al. (2021) and Stefano (2024) underscored the importance of PET texture features in capturing metabolic heterogeneity and tumor biology. These findings suggest a complementary relationship between CT and PET radiomics features (Du et al. 2021; Lv et al. 2021; Stefano et al. 2021).Importantly, clinical variables such as nodal (N) stage, gender, histology, and lymphovascular invasion consistently ranked among the top contributors, underscoring that staging context and pathological risk factors remain essential to reliable prediction. This synergy suggests that while CT radiomics provides strong baseline prognostic signal, PET enriches the model with metabolic information, and clinical variables anchor predictions to established risk factors—together enabling more comprehensive and clinically interpretable stratification.

## Limitations

Several limitations merit consideration. First, the cohort was modest (n = 131) and from a single centre, which may limit generalisability. Second, the retrospective design and class imbalance warrant caution, and external validation was not undertaken. Third, we relied on hand-crafted radiomics with classical machine-learning models; integrating deep-learning features into interpretable fusion architectures could improve performance while maintaining transparency. Future work will prioritise multicentre validation with harmonised acquisition protocols, time-to-recurrence modelling rather than a binary endpoint, and prospective evaluation of decision-support tools for adjuvant therapy selection and surveillance planning to facilitate clinical translation.

## Conclusion

In this study, we systematically evaluated machine learning models for predicting postoperative recurrence in NSCLC using CT, PET, and clinical data, both individually and in combination. Multimodal integration—particularly late fusion of PET, CT, and clinical features with ElasticNet—showed improved discriminatory performance, more stable calibration, and potential clinical net benefit. Fusion models also appeared to support broader identification of high-risk patients and more consistent risk stratification, which may help guide surveillance schedules, adjuvant therapy decisions, and prognostic discussions. Overall, our findings support the value of moving beyond TNM staging or single-modality models toward integrated, interpretable multimodal frameworks that, with further validation in larger multi-center cohorts, could contribute to more practical tools for precision oncology in NSCLC.

## Supplementary Information

Below is the link to the electronic supplementary material.

Supplementary Material 1

## Data Availability

The datasets analysed in this study are publicly available from the NSCLC-Radiogenomics dataset hosted on The Cancer Imaging Archive (TCIA). [NSCLC-RADIOGENOMICS - The Cancer Imaging Archive (TCIA)] (https://www.cancerimagingarchive.net/collection/nsclc-radiogenomics) .
