# Supplementary material for: Multimodal radiomics fusion for predicting postoperative recurrence in NSCLC patients
Source: J Cancer Res Clin Oncol. 2025 Sep 18;151(10):261. doi: 10.1007/s00432-025-06311-w (PMC12446158; doi:10.1007/s00432-025-06311-w)
Supplement: Supplementary file 1 — Supplementary Material 1 [file 432_2025_6311_MOESM1_ESM.docx]

**Supplementary Table S1**. Patients' demographic and clinicopathological characteristics

| **Variables** | **Full Cohort (n=131)** |
| --- | --- |
| **Recurrence Proportion** | 29.0% (38/131) |
| **Age (years)** | 69.1 ± 8.6 |
| 0–50 | 4 (3.1%) |
| 50–60 | 11 (8.4%) |
| 60–70 | 62 (47.3%) |
| 70–80 | 42 (32.1%) |
| 80–100 | 12 (9.2%) |
| **Gender** |  |
| Female | 31 (23.7%) |
| Male | 100 (76.3%) |
| **Weight (kg)** | 172.0 ± 39.1 |
| <50 | 0 (0.0%) |
| 50–60 | 0 (0.0%) |
| 60–70 | 0 (0.0%) |
| 70–80 | 1 (0.8%) |
| >80 | 130 (99.2%) |
| **Pack Years** | 35.2 ± 29.8 |
| 0 | 24 (18.3%) |
| 0–10 | 17 (13.0%) |
| 10–20 | 10 (7.6%) |
| 20–30 | 13 (9.9%) |
| 30–40 | 14 (10.7%) |
| 40+ | 50 (38.2%) |
| **Ethnicity** |  |
| African-American | 5 (3.8%) |
| Asian | 20 (15.3%) |
| Caucasian | 98 (74.8%) |
| Hispanic/Latino | 5 (3.8%) |
| Native Hawaiian/Pacific Islander | 3 (2.3%) |
| **T Stage** |  |
| T1a | 33 (25.2%) |
| T1b | 28 (21.4%) |
| T2a | 36 (27.5%) |
| T2b | 9 (6.9%) |
| T3 | 16 (12.2%) |
| T4 | 4 (3.1%) |
| Tis | 5 (3.8%) |
| **N Stage** |  |
| N0 | 104 (79.4%) |
| N1 | 11 (8.4%) |
| N2 | 16 (12.2%) |
| **M Stage** |  |
| M0 | 127 (96.9%) |
| M1a | 1 (0.8%) |
| M1b | 3 (2.3%) |
| **Grade** |  |
| G1 Well Differentiated | 24 (18.3%) |
| G2 Moderately Differentiated | 62 (47.3%) |
| G3 Poorly Differentiated | 27 (20.6%) |
| Other Type I (Well–Moderately) | 9 (6.9%) |
| Other Type II (Moderately–Poor) | 9 (6.9%) |
| **Smoking Status** |  |
| Current | 27 (20.6%) |
| Former | 85 (64.9%) |
| Nonsmoker | 19 (14.5%) |
| **Histology** |  |
| Adenocarcinoma | 103 (78.6%) |
| NSCLC NOS | 3 (2.3%) |
| Squamous Cell Carcinoma | 25 (19.1%) |
| **Lymphovascular Invasion** |  |
| Absent | 113 (86.3%) |
| Present | 18 (13.7%) |
| **Pleural Invasion** |  |
| No | 94 (71.8%) |
| Yes | 37 (28.2%) |
| **High Risk 2yr** |  |
| No | 105 (80.2%) |
| Yes | 26 (19.8%) |
| **Survival Time (days)** |  |
| Mean ± SD | 1092.1 ± 781.9 |
| Median [IQR] | 1062.0 [300.0–1826.5] |
| Range | 6 – 3074 |

Continuous variables (RobustScaler applied): Age, Weight, Pack_Years, T_stage, N_stage, M_stage, Grade, Survival_Time. Binary/Categorical variables (no scaling): All Gender_*, Ethnicity_*, Smoking_status_*, Histology_*, Lymphovascular_invasion, Pleural_invasion, Event, High_Risk_2yr. Analysis method: Nested Cross-Validation on full cohort. Cross-validation: 3-fold outer CV, 2-fold inner CV for hyperparameter optimization. Class balancing: SMOTE applied within CV folds. Missing data: Handled by KNN/Simple imputer within pipeline.
